# Prospects and Promises

**Published:** 1917-12-29

**Authors:** 


### The Hospital

The Workers' Newspaper of Administrative Medicine and Institutional Life, Administration, National Insurance and Health.

No. 1647, Vol. LXIII. SATURDAY, DECEMBER 29, 1917. PRICE ONE PENNY

### PROSPECTS AND PROMISES.

The end of the year, appropriately and naturally, is in normal circumstances associated rather with memories than with anticipations. The record of a definite time period is written, and the end of the period suggests that the record be reviewed. Out of the review may come, it is true, lessons and guidance for the future, but the tendency of the mind at the closing stage is to look at the experiences and incidents which have marked the course of the movement. Good or bad, or the two in various proportions, they come to the surface with the termination of the year which has included their history. The present time, however, is not a normal one. It is crowded with events so great in themselves and so pregnant with issues for the future that almost insensibly our look is directed forward rather than backward. To examine the past needs some effort of the will and an ability to shut oneself off from the tension and pressure of the hour. The history of an age has been closed by the outbreak of a world-shaking war, and a new era is about to commence. The character and destiny of this new era are being shaped by events which force themselves on our daily attention, and all the attraction of the hour is towards the new world which is to be born. Thus, even at the close of the year, the disposition of men's minds is directed to what is to come, and the eager look of most of us is turned on the New Year. There is a general consciousness that we have soon to face experiences different from any which have hitherto fallen to our lot, and from this conviction naturally arise wonder and anticipation. The past is beyond us, and it is, moreover, separated in kind from the experiences which await us. Over it we may not linger, for more than ever before we have at hand a future filled with a sense of the unknown. The range of this unknown appears enormous. Not only for individuals or limited communities does the uncertainty exist. Rather the fate of the whole world trembles in the balance, and there is forced on our attention the question whether or not freedom shall perish from the earth. Here is the commanding consideration of the day and hour, and this it is which turns men's eyes towards the horizon and leads them to scan with keen vision the promise and prospect of the new day.

In these columns, as our readers are aware, we profess to be continuously and specially interested in all that concerns the stability and development of our great voluntary hospital system, and it is in the very nature of things that we should speculate and wonder what the future has in store for the organisations and agencies which compose it. The struggle which at the present moment is holding the world in desolation is not merely a military and a political issue. Nor is it only a war of nations in the numerical and mechanical meaning of this term. It obviously includes these elements, but it includes also something more. It is an affair of the mind and spirit and outlook on life, and opposing and antagonistic conceptions are engaged in its conduct and aim. The victory will be not only the victory of an army, and not only the victory of a people. It will be the triumph of an idea and will mark the whole range of the future, and this for many ages yet to come. There is a commanding word to be spoken, and the notes of it will be heard in every department of human activity and human thought. On the one side is set the determination to compel men to schemes of life arranged and ordered by more or less arbitrary authorities; on the other is the claim for self-organisation and for the liberties and freedom of choice of the people. Here is the region in which in essence the battle of ideas is joined, and the victory on one side or the other will be complete. There is no compromise, and to look for a half-way house were a vain search. The question does not admit of a divided or ambiguous answer, and every man must elect to stand or fall on the decision. It is because of this spiritual aspect of the struggle that the contest commands the future and forces attention on what this is to bring. Our own nation from the outset made its choice, and in spite of disappointments and losses and griefs which may not be spoken there is not, so far as we can judge, any weakening of the people's will. In some instinctive fashion there is realised that we stand at a supreme day, not only in our own history but in the history of civilisation, and that by the most sacred of obligations we are compelled to make good our cause. Such a consideration dominates all other debate, and we know without reasoning that it must be supreme in our national life.

As we turn to the special interests and activities with which we are here mainly concerned we find abundant reason for hope and confidence. That the national cause will triumph we make no doubt, seeing it is founded on principles which are rooted in the eternal fitness of things and which are essential to human dignity and to the welfare of man's estate. What makes a particular appeal to us is that this good cause rests so largely on personal service and voluntary effort. On the broadest lines we see nations apparently remote from the material contest willing and ready to stake their all in order that freedom may be secured for the small as for the great peoples. Such an announcement is set in large letters, which must rest for ever conspicuous in human history. Coming to a less public platform, we may see in our own country and national organisation enterprises for the care and welfare of our soldiers carried by voluntary workers to an efficiency and tenderness of service quite beyond the capacity of a rigid governing authority. Of some of these the praise is on all our lips, but the spirit is the same in the multitudes of obscure and hidden activities the very existence of which is unknown beyond the small towns and villages where their merciful mission displays itself. Never before have the dignity and duty of voluntary effort and personal service spread so widely or commanded so large a triumph. Here is, we venture to say, a commencing triumph of the great idea for which the manhood of our race stand to-day in arms, and in the guarantee of this promise we look to the future with courageous and unyielding confidence.

